# Tuning pore size and density of rigid polylactic acid foams through thermally induced phase separation and optimization using response surface methodology

**DOI:** 10.1038/s41598-024-62958-4

**Published:** 2024-05-29

**Authors:** Morteza Ghorbandoust, Mohammad Fasihi, Reza Norouzbeigi

**Affiliations:** School of Chemical, Petroleum and Gas Engineering, Iran University of Science and Technology, Mail Box: 16846-13114, Narmak, Tehran Iran

**Keywords:** Foam, Phase separation, Polylactic acid, Response surface, Morphology, Materials science, Biomaterials, Soft materials

## Abstract

Rigid polylactic acid (PLA) foams fabricated via thermally induced phase separation (TIPS) utilizing a ternary solution of PLA, Tetrahydrofuran (THF), and water. The PLA gels were stabilized mechanically by the substituting of the THF/water solvent mixture with ethanol as non-solvent and subsequently vacuum dried. A comprehensive characterization of PLA foams was achieved by Scanning Electron Microscopy (SEM), X-ray Diffractometry (XRD) and Brunauer–Emmett–Teller (BET) analyses. The BET area obtained in the PLA foam is up to 18.76 m^2^/g. The Response Surface Methodology (RSM) was utilized to assess the impacts of four independent variables (polymer concentration, solvent composition, quench temperature, and aging time) on the pore size and density of PLA foam. The experimental findings demonstrated that the fabrication parameters could be fine-tuned to govern the morphology of the pores, comprising their size and density. The optimal values of parameters for cell size were identified by RSM to be 8.96 (wt%), 91.60 (w/w), 5.50 °C, and 3.86 h for the optimum cell size of 37.96 µm (37.78 by Genetic Algorithm). Optimum density by RSM 88.88 mgr/cm^3^ (88.38 mgr/cm^3^ by Genetic Algorithm) was obtained at 5.00 (wt%), 89.33 (w/w), 14.40 °C and 2.65 h.

## Introduction

Polylactic acid, also known as PLA, is a highly utilized biopolymer that holds a significant share of 33% among all bioplastics manufactured in the year 2021. PLA is derived from natural and renewable resources, possessing favorable physical and chemical characteristics. Moreover, it undergoes biodegradation within specific environmental conditions of temperature, pH, and moisture levels, typically found in composting process^1^.

Numerous scientific inquiries have been carried out to examine the process of biodegradation in polylactic acid and its mixtures with various polymers. In a study by Litauszki et al.^2^, it was demonstrated that foam sheets based on polylactic acid containing a higher proportion of D-lactide exhibited a faster decomposition rate, with the foam sheet decomposing in 49 days. In contrast, foam sheets with a lower D-lactide content took longer to decompose, requiring 63 days for complete degradation, when an 8 wt% foaming agent was utilized. A study conducted by Auras et al.^3^ demonstrated PLA can be effectively hydrolyzed through the application of heat. Boiling water or steam can be used to break down the polymer, resulting in the formation of lactic acid that recycled and utilized as a monomer, allowing for the new production. By subjecting PLLA to hydrolysis at temperatures ranging from 180 to 350 °C for a duration of up to 30 min, researchers were able to obtain L-lactic acid as the final product. This demonstrates the feasibility of molecular recycling and highlights the potential for a more sustainable approach to material reuse.

PLA has been reported to break down into CO_2_ and water within a period of less than 90 days in a specific composting situation. This process occurs in what is known as a "controlled composting environment," where the conditions are carefully regulated to facilitate the decomposition of PLA. The controlled composting environment provides the necessary conditions for the accelerated degradation of PLA, allowing it to be broken down efficiently by a diverse microbial population. However, it is essential to note that the degradation process of polylactic acid necessitates a considerably high temperature range of 55–175 °C, which surpasses the natural environmental conditions^4^.

Narancic et al.^5^ conducted research on the biodegradation of polylactic acid and various polymers, as well as their blends, by employing biodegradation standards. The study findings revealed that polylactic acid did not undergo degradation within a 56-day experiment conducted in a simulated aquatic environment. The experiment took place in anaerobic sludge obtained from a wastewater treatment plant, under controlled conditions of 35 ± 2 °C. Bagheri et al.^6^ conducted research on the decomposition of polylactic acid, as well as various biopolymers and synthetic polymers, in simulated freshwater and seawater environments. The study took place in a controlled thermostatic chamber set at 25 °C, with a light cycle of 16 h of fluorescence light followed by 8 h of darkness. Through their experiment lasting 400 days, the researchers observed that polylactic acid exhibited minimal degradation compared to the other materials tested.

PLA has a broad spectrum of applications, such as packaging, structural foams, housewares, thermal and acoustic insulation, medical and pharmaceutical purposes, filtration and separation^7^. In contrast to petroleum-based polymers, the manufacturing process of PLA consumes notably less energy, with a reduction of 25 to 55%^8–10^. Various procedures for the regulated production of polymeric foams have been established, such as electrospinning, particulate leaching, fiber bonding, Sol–gel, gas foaming and phase separation^5^.

TIPS is an extensively used technique to produce scaffolds and porous foam purposes. The procedure involves preparing a polymer solution that exhibits upper critical solution temperature (UCST) characteristics by heating it to elevated temperatures. The procedure involves the gradually reduction of temperature in a homogeneous polymeric solution until it reaches a critical point where the single-phase system becomes thermodynamically unstable, resulting in a spontaneous separation into two distinct phases: a polymer-poor and polymer-rich phase. Generally, the utilized system comprises a ternary solution consisting of a polymer, a solvent, and a non-solvent. The addition of the non-solvent augments the free energy of mixing, which in turn facilitating the process of phase separation^12,13^.

**Figure 1:**
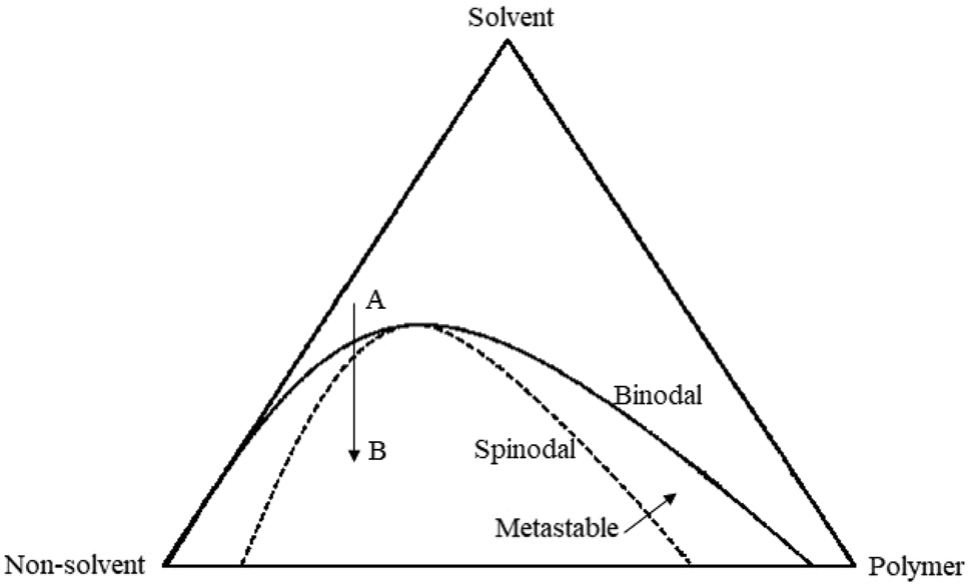
Ternary phase diagram of a polymer/solvent/non-solvent system.

The resulting foam morphology is directly influenced by the specific thermodynamic conditions present during the quenching of the solution. As shown in Fig. 1, the polymer solution can enter two-phase regions when it is quenched from point A to point B by reducing the temperature at a specific concentration. In the event of entering metastable regions, the separation of phases is expected to progress through the mechanism of nucleation and growth. In the unstable areas, the process of phase separation follows the mechanism known as spinodal decomposition^14^.

In the metastable area, the phenomenon of phase separation occurs through a complex process involving nucleation and growth. This process is driven by instantaneous thermal and compositional fluctuations, which result in the formation of tiny droplets of the new phase. The droplets expand as a result of solute diffusion and ultimately merge together. After the formation of the droplet, the local surroundings will encounter solute depletion, leading to the restriction of droplet growth due to molecular diffusion across the concentration gradient.

**Figure 2:**
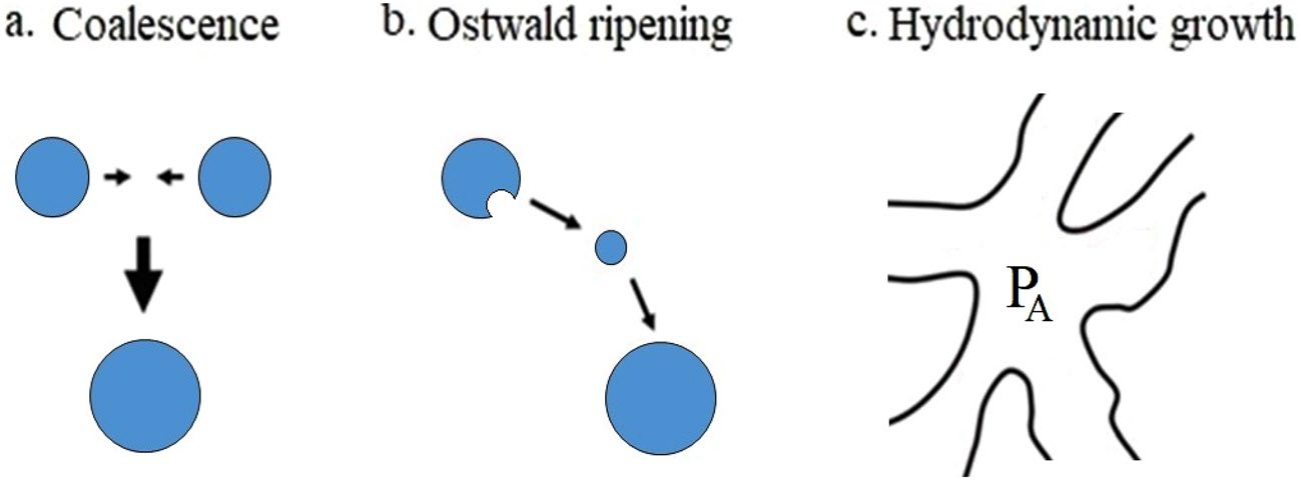
Illustration of the three coarsening mechanisms, (**a**) coalescence, (**b**) Ostwald ripening, and (**c**) hydrodynamic growth.

In the unstable region, the lack of a kinetic barrier against the process of phase separation enables the occurrence of spinodal decomposition, leading to the formation of a bicontinuous network. This network is particularly susceptible to the presence of a polymer lean phase, resulting in a high interfacial energy that can be reduced through the implementation of coarsening mechanisms, such as droplet coalescence, Ostwald ripening, and hydrodynamic growth (Fig. 2)^15,16^. Before attaining the minimum energy state, the phase separation process can be impeded by the gelation or crystallization of the polymer-rich phase. This results in the kinetic arrest of the system, preventing it from fully separating into distinct phases^17^.

After the phase separation process has been completed or stopped, the solvent can be eliminated using three different methods: freeze drying, supercritical drying, or replacement with a non-solvent and subsequent vacuum drying.

Zanotti et al.^18^, carried out a research project to examine the effects of freeze-drying and SC-CO_2_ drying on the structure and morphology of a porous scaffold. The findings of their research revealed that the gel morphology obtained through SC-CO_2_ drying exhibited an open and regular structure. In contrast, the freeze-dried gels displayed a collapsed and irregular structure at the microscale, and were completely collapsed at the nanoscale. SC-CO_2_ offers a promising alternative for processing polymers without compromising their structural integrity. By utilizing its zero surface tension and gas-like diffusivity, SC-CO_2_ can effectively interact with polymer matrices without causing collapse. On the other hand, Freeze-drying treatment, while commonly used, can result in structural disorder due to the formation and removal of ice crystals during the drying process.

Additionally, the significant difference in specific surface areas between supercritically dried gels and freeze-dried gels highlights the importance of the drying method in determining the final characteristics of the gel material. Conversely, certain studies have indicated that a rapid depressurization rate of SC-CO_2_ could result in shrinkage and cracking, particularly if the rate is excessively high. In certain instances, carbon dioxide may bypass the gels within vessels, resulting in incomplete drying and subsequently leading to the formation of cracked gels. This incomplete drying process may be attributed to the inadequate displacement of water from the gel structure during supercritical drying^19,20^.

Besides, Freeze-drying, a procedure that may extend from 3 to 7 days, is known for its high energy consumption. The disadvantages of supercritical drying methods revolve around the challenging temperature and pressure conditions, along with the discontinuous operational approach. Furthermore, freeze drying and supercritical drying have a negative effect primarily on appearance and require grueling processes that in turn can lead to textural damage^21^. Solvent exchange is a straightforward process that does not require sophisticated equipment.

To obtain a homogeneous foam, it is crucial to execute a non-solvent substitution after the finalization of phase separation. To prevent drying shrinkage caused by capillary action, the solvent mixtures were replaced with non-solvent prior to the start of vacuum drying. In this section, the solvent was extracted by slowly pulling back the plunger of syringe tube and replaced by ethanol. This process was repeated further one more time. If neglected, the presence of solvent can lead to an uncontrolled alteration in the end result^9^.

The Design of Experiment (DOE) method is considered a reliable approach for investigating and predicting interactions among phase separation parameters. This method utilizes linear regression and Analysis Of Variance (ANOVA) to analyze the interaction between a set of selected parameters. Additionally, the DOE design enables the estimation of a test model, which provides valuable equations for accurately calculating anticipated output values in advance^22,23^. Through a review of the current evidence, the main objective of this investigation was to study the effect of the polymer concentration, solvent composition, quench temperature, and aging time on morphology and density of the polylactic acid foams (response)^24–28^. Moreover, extra aim of this examination was to determine the optimal values of the crucial factors, known as the TIPS variables, using RSM. Unexpectedly, there is no evidence in the literature regarding about manipulation of aging time in the solvent exchange technique. This lack of scholarly investigation into the regulation of aging time a significant challenge in comprehending the optimal control mechanisms for this specific procedure^11^. So, for the first time, we studied aging time effect on the foam morphology and density. Another key goal of this research is to utilize TIPS methodology to design an open cell PLA foam for the purposes of separation and sorption of some material such as oil–water solutions, dyes, and heavy metals from aqueous solution. The current article focuses solely on the influence of processing variables on the morphology and density of the foams produced, serving as the initial stage of a more comprehensive study. The second part of the project will emphasize the critical significance of this element, as it involves the design of a biocompatible open cell foam using PLA as the base material with a customized cell size and a density. As a result, another novel aspect of the current investigation is the establishment of suitable combinations of processing parameters required for determining the optimal cell size and foam density for separation and sorption processes.

## Materials and experiment

### Materials

The PLA employed in this specific investigation was a commercial grade 4042D. It has 4% of D-isomer and density of 1.24 g/cm^3^, supplied by NatureWorks (USA). Tetrahydrofuran (THF) and Ethanol were bought from Merck. Double-distilled deionized water was consistently utilized throughout the entirety of the experiments.

### Experimental design

The evaluation of multiple dependent variables in experimental work makes the traditional method both costly and time consuming. To address this issue, a multivariable analysis technique was utilized in the design of experiments (DOE) to define the effects and interactions of independent variables on the result of thermally induced phase separation (TIPS). This approach aims to streamline the process and provide a more efficient means of studying the phenomenon. Several forms of Design of Experiments (DOE) are available, each offering unique methodologies and approaches, screening DOE, fractional factorials, full factorials, RSM and Taguchi designs^29^. RSM comprises a range of mathematical and statistical tools that play a crucial role in the development, improvement, and optimization of various processes. RSM provides a faster and more practical method to optimize complex processes. This methodology will not only determine optimal conditions based on a limited number of trials, but it will also provide the necessary knowledge to evaluate results in order to develop a procedure. The utilization of analysis of variance (ANOVA) is an indispensable procedure in ascertaining the adequacy, significance and meaningfulness of the developed model^30^. This technique has been effectively employed to optimizing of PLA-PCL electrospun fiber^31^ LLDPE microporous membrane^32^, PLA foaming^10^.

The objective of this study is to explore the impacts of the four variables as mentioned above, on the morphology and density of PLA foams. The range of variables based on the variables used in previous studies were: PLA concentration (y_1_) from 5 to 9 (%w), THF/water ratio (y_2_) from 84 to 92 (w/w), temperature (y_3_) from − 5 to 35 °C, and aging time (y_4_) from 2 to 10 h.

The independent variables, both coded and uncoded, that were employed in the RSM design were documented in Table 1. The effects of individual parameters and their interactions were explored using a quadratic model integrated with a central composite rotatable design (CCRD). A total of 31 experimental results are summarized in Tables 2, 3. The CCRD technique involved conducting repeatability tests seven times at the central point. The acquired data underwent analysis through multiple regressions utilizing the least-square method. This approach offers the comprehensive justification for the positioning of the line of best fit among the data points under examination. The linear relationship between the cell size and density (response) and the independent variables as follows:

1 $${R}_{x}={\gamma }_{0}+\sum_{i=1}^{4}{\gamma }_{i}{y}_{i}+\sum_{i=1}^{4}{\gamma }_{ii}{{y}_{i}}^{2}+\sum_{i=1}^{3}\sum_{j=i+1}^{4}{\gamma }_{ij}{y}_{i}{y}_{j}$$

where R_x_ is the predicted response, γ_0_ is the constant, y_i_ and y_j_ are the levels of independent variables, γ_i_, γ_ii_ and γ_ij_ are the linear, quadratic and interaction terms, respectively^33^.

**Table 1:** Coded and uncoded levels of independent process variables for cell size and density.

Coded level	y_1_ (wt%)	y_2_ (w/w)	y_3_ (°C)	y_4_ (h)
− 2	5	84	− 5	2
− 1	6	86	5	4
0	7	88	15	6
+ 1	8	90	25	8
+ 2	9	92	35	10

**Table 2:** The CCRD and experimental data for cell size.

Run order	y_1_ (wt%)	y_2_ (w/w)	y_3_ (°C)	y_4_ (h)	Size (µm)	St. dev (± µm)
1	6	90	5	8	106.23	7.49
2	7	88	15	6	92.09	23.98
3	8	90	5	8	62.96	10.36
4	7	88	15	6	77.23	8.88
5	5	88	15	6	189.18	73.89
6	8	86	25	8	142.05	25.53
7	7	88	15	6	87.08	17.89
8	6	90	25	8	140.06	5.09
9	6	86	5	4	125.03	13.57
10	9	88	15	6	81.85	13.79
11	7	88	35	6	118.54	8.03
12	6	86	25	8	158.31	17.12
13	6	86	25	4	134.22	19.28
14	7	88	15	2	92.57	12.95
15	8	86	25	4	90.53	31.75
16	7	88	15	6	92.04	17.71
17	8	90	25	8	90.21	13.72
18	7	88	− 5	6	89.32	42.29
19	6	90	25	4	117.44	18.13
20	7	88	15	6	76.29	14.99
21	7	88	15	6	84.65	20.79
22	6	90	5	4	134.53	15.77
23	8	90	25	4	55.11	10.70
24	8	90	5	4	39.04	12.47
25	6	86	5	8	142.18	17.00
26	8	86	5	4	64.14	29.79
27	7	84	15	6	115.04	22.82
28	7	92	15	6	92.25	49.45
29	7	88	15	10	120.74	18.80
30	8	86	5	8	105.16	29.20
31	7	88	15	6	71.19	19.52

**Table 3:** The CCRD and experimental data for density.

Run order	y_1_ (wt%)	y_2_ (w/w)	y_3_ (°C)	y_4_ (h)	Density (mg/cm^3^)	St. dev (± mg/cm^3^)
1	6	90	5	8	145.02	7.25
2	7	88	15	6	121.08	6.05
3	8	90	5	8	201.34	10.06
4	7	88	15	6	111.41	5.57
5	5	88	15	6	93.06	4.70
6	8	86	25	8	119.52	5.97
7	7	88	15	6	122.59	6.13
8	6	90	25	8	118.04	5.90
9	6	86	5	4	129.63	5.98
10	9	88	15	6	170.23	8.51
11	7	88	35	6	112.49	5.62
12	6	86	25	8	127.46	5.87
13	6	86	25	4	111.07	5.90
14	7	88	15	2	112.95	5.64
15	8	86	25	4	135.25	6.76
16	7	88	15	6	117.87	5.89
17	8	90	25	8	155.18	7.75
18	7	88	− 5	6	170.09	8.50
19	6	90	25	4	108.63	5.43
20	7	88	15	6	120.09	6.00
21	7	88	15	6	109.27	5.46
22	6	90	5	4	124.60	6.23
23	8	90	25	4	135.32	6.76
24	8	90	5	4	163.98	8.19
25	6	86	5	8	160.82	6.89
26	8	86	5	4	157.83	7.89
27	7	84	15	6	158.71	7.93
28	7	92	15	6	160.74	8.03
29	7	88	15	10	148.37	7.41
30	8	86	5	8	176.83	8.84
31	7	88	15	6	110.78	5.53

### Foam preparation

The first step involved the preparation of PLA/THF/water solutions by dissolving the polymer in the solvent (according the ratio in Table 1). This dissolution process was facilitated by stirring the mixture at 500 rpm and maintaining a temperature of 60 °C.

Raising the temperature of the solution led to the formation of more thermally stable crystallites due to the increased chain mobility at higher temperatures. This allowed the polymer chains within the precipitate to rearrange into a more stable configuration, resulting in a higher level of crystallinity^34^. Increasing the degree of crystallinity also resulted in a higher material density, which may not be desirable in certain applications. It is crucial to note that the solution temperature should not exceed the boiling point of THF, which is 65.81 °C at atmospheric pressure. This limitation is important to prevent the solvent from boiling off during the heating process, which could negatively impact the formation of crystallites in the polymer. Maintaining the solution temperature below this threshold ensures that the polymer chains have sufficient time to reorganize and form stable crystalline structures without the risk of solvent loss^35^.

In their study, Mahmoudi et al.^36^ made an interesting observation regarding the impact of stirrer speed on the morphology of particles. They found that the speed of the stirrer had a dual effect on the particles' characteristics. Firstly, when the speed was increased, it led to a reduction in particle size. This suggests that higher stirrer speeds can contribute to the shrinking of particle dimensions. However, the researchers also noted that increasing the stirrer speed had another consequence. It caused the formation of larger air bubbles, indicating that higher speeds can promote the generation of these bubbles within the system. In a separate investigation, Riphatin et al.^37^ focused on the physical structure of polyurethane foam and its relationship with mixing speed. Their findings revealed that a well-defined physical structure of the foam was achieved when the mixing speed reached 500 rpm. This suggests that a specific mixing speed is crucial for obtaining the desired physical characteristics of foam.

The solution was deemed clear when the polymer had completely dissolved. The high porous foams were achieved by incorporating a range of 5 to 9 wt% of PLA.

The obtained solution was carefully poured into a Pyrex test tube, which had dimensions of 100 by 10 mm. To induce phase separation, the samples were subjected to five different quenching temperatures within a storage range of − 5 to 35 °C. The selection of these cooling temperatures was made to enforce varying cooling rates, resulting in distinct crystallization conditions^38^.

**Figure 3:**
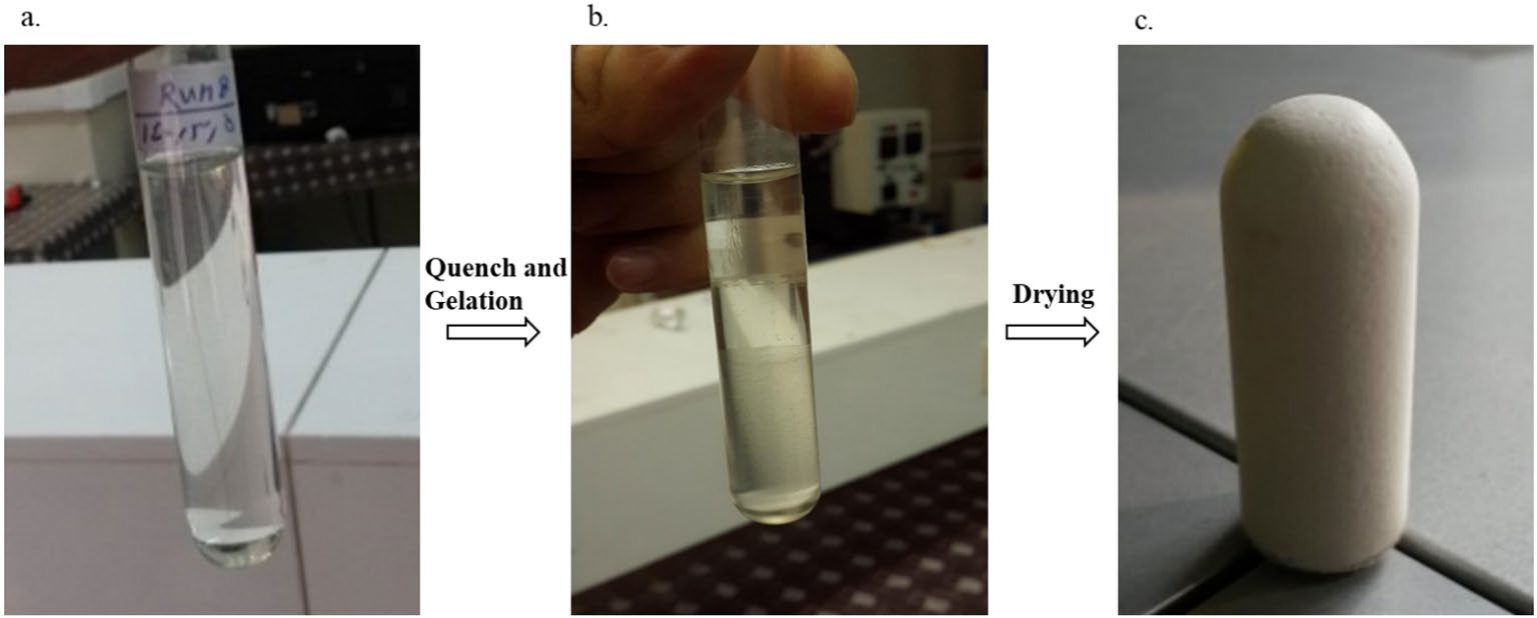
PLA foam fabrication steps, (**a**) polymer solution, (**b**) quench and gelatin, and (**c**) foam obtained after vacuum drying.

Upon completion of the quenching step, the test tubes, which contained PLA gels, were refilled with ethanol twice in aging time durations. The obtained foams were removed from the test tubes and dried in a vacuum oven at 35 °C until a consistent weight was achieved. Subsequently, all samples were stored in sealed vials within a desiccator for additional analysis. Figure 3 illustrations a schematic representation of the various stages of the foaming production.

### Testing and characterization

**Figure 4:**
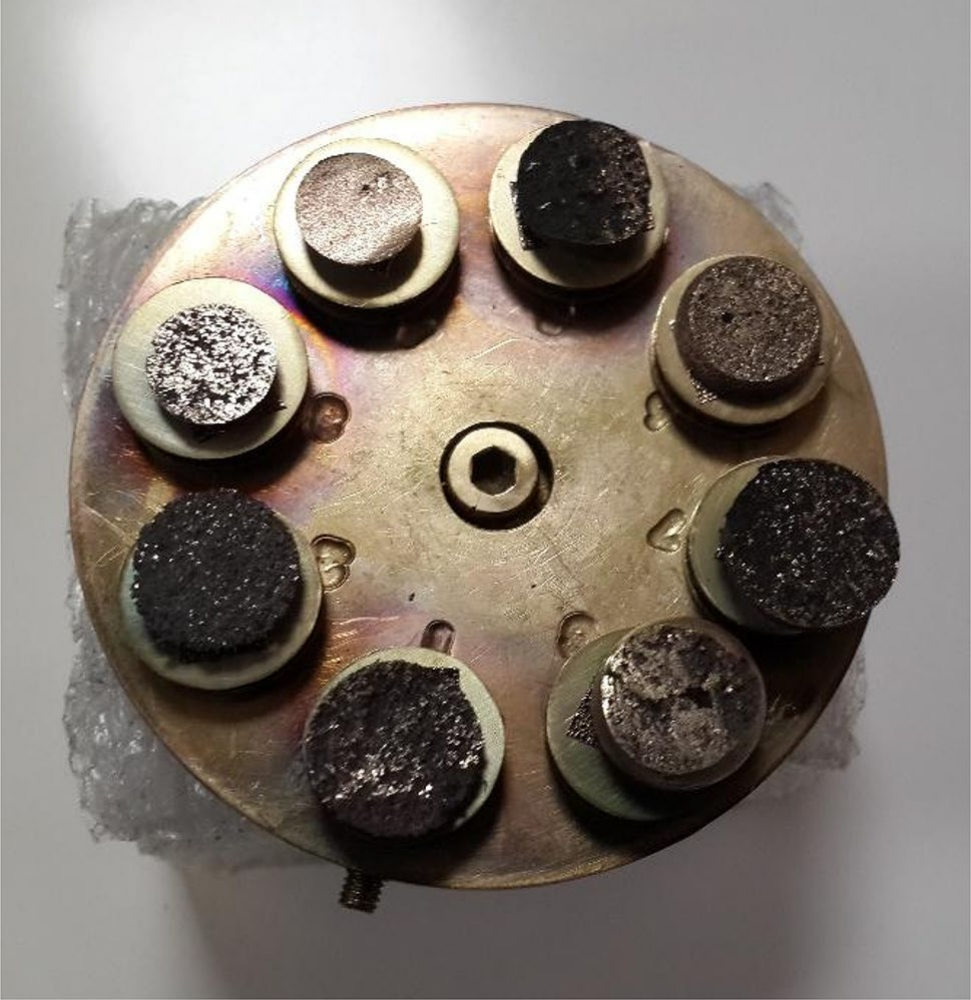
Cross-sections of prepared foams after gold coating.

The Brunauer-Emmette-Teller (BET) N_2_ adsorption–desorption test (BELSORP II MINI) is employed to determine the specific surface area of the samples at a temperature of 77.3 K, following a degassing process. The morphology of the foams was studied using scanning electron microscopy (SEM; VEGA II, TESCAN) in secondary electron mode operation. In order to avoid any problems with charging while conducting the analysis, the cross-section of the samples was coated with a layer of gold through a sputter-coater (Desk Sputter Coater, DSR1). A cross-section was obtained by cutting through a material with a razor blade perpendicular to its main axis (Fig. 4). The coating process involved setting the Ar gas pressure to 5 psi and maintaining a current of 10 mA for a duration of 5 min.

The foams' crystallinity percentage was assessed using X-ray diffractometry (XRD). Diffraction patterns were generated using a Bruker D8-Advance X-ray diffractometer (Bruker, Germany), which featured a detector across a 2θ range from 5 to 90° and functioning at 40 kV and 40 mA. The average cell size was determined by analyzing of at least 50 cells for each creation^39^. To calculate the foam densities, the weight of the foams was divided by their volume. The specific volume was calculated via Eq. (2), where ρ and ρ_f_ are the density of PLA and PLA foam, respectively. The porosity (ɛ) of foam can be achieved by Eq. (3)

2 $${v}_{s}=\frac{{\rho }_{f}}{\rho }$$

3 $$\upvarepsilon =1-{v}_{s}$$

## Result and discussion

### PLA foam morphology

Polylactic acid polymers can exist in different structural forms, including amorphous and glassy polymers, as well as semi-crystalline and highly crystalline polymers. When a solution of polymer exhibiting UCST is subjected to cooling, Polymer crystallites that are created during the phase separation process are capable of physically interconnecting, and this ability is contingent upon the concentration of the solution. Polymer-rich phase gelation is a consequence of the phenomenon as mentioned above, a gel-like structure is created through the progression of this process. The occurrence of liquid–liquid phase separation (LLPS) leads to the generation of a network-like structure consisting of a polymer-rich phase containing both the polymer and a certain amount of the solvent, and a polymer-poor phase composed of the non-solvent and the remaining solvent. The experimental parameters employed during the TIPS process had an impact on the final morphology of the foam. These parameters include the mechanism of the coarsening process, phase separation, and the gelation of the polymer-rich phase. The gelation phenomenon serves as a limiting factor for the coarsening process, effectively halting any further development in the morphology of the foam.

**Figure 5:**
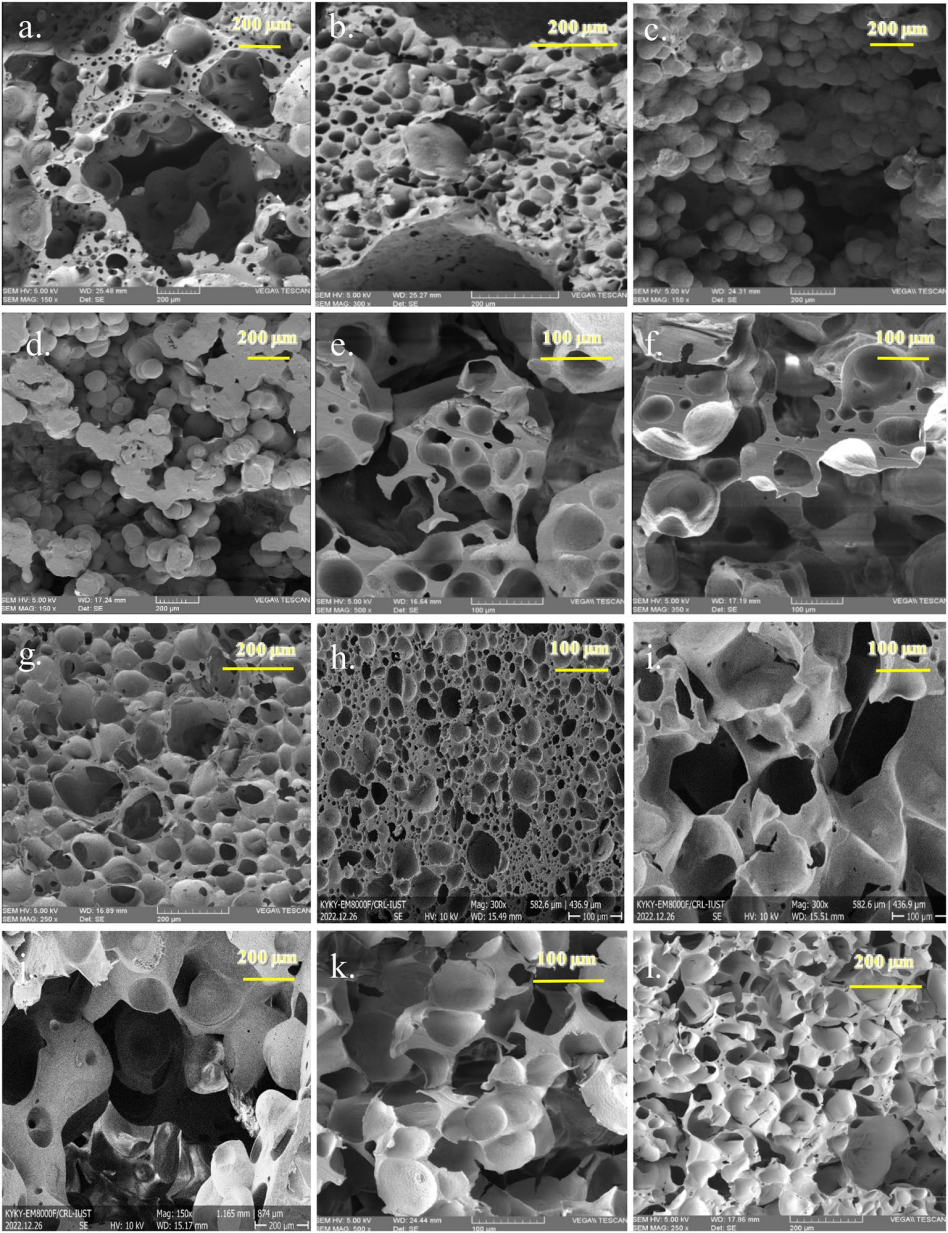
SEM images of fractured surface of foams: (**a**) Run 5, (**b**) Run 9, (**c**) Run 10, (**d**) Run 11, (**e**) Run 12, (**f**) Run 15, (**g**) Run 18, (**h**) Run 24, (**i**) Run 26, (**j**) Run 28, (**k**) Run 29, (**l**) Run 30.

Figure 5, 6 show cross-section SEM images and the corresponding pore size distribution (PSD) curves for different polylactic acid (PLA) foams. The evaluation of pore characteristics, such as pore size, and pore size distribution in the foam samples' cross-sections, was conducted using ImageJ software. The cellular structure of the PLA foam exhibited a significant irregularity, comprising both micro and macro bubbles. The cell size distribution was narrow, and the diameter of cells was approximately between 39 and 189 µm. In the PSD analysis of all produced foam samples, it is evident that a reduced polymer concentration (5 wt%) leads to a broader PSD when contrasted with a solution containing a higher polymer concentration (9 wt%). The standard deviation of the cell size distribution was measured. This factor measures how far the average value lies from the mean^40^.

**Figure 6:**
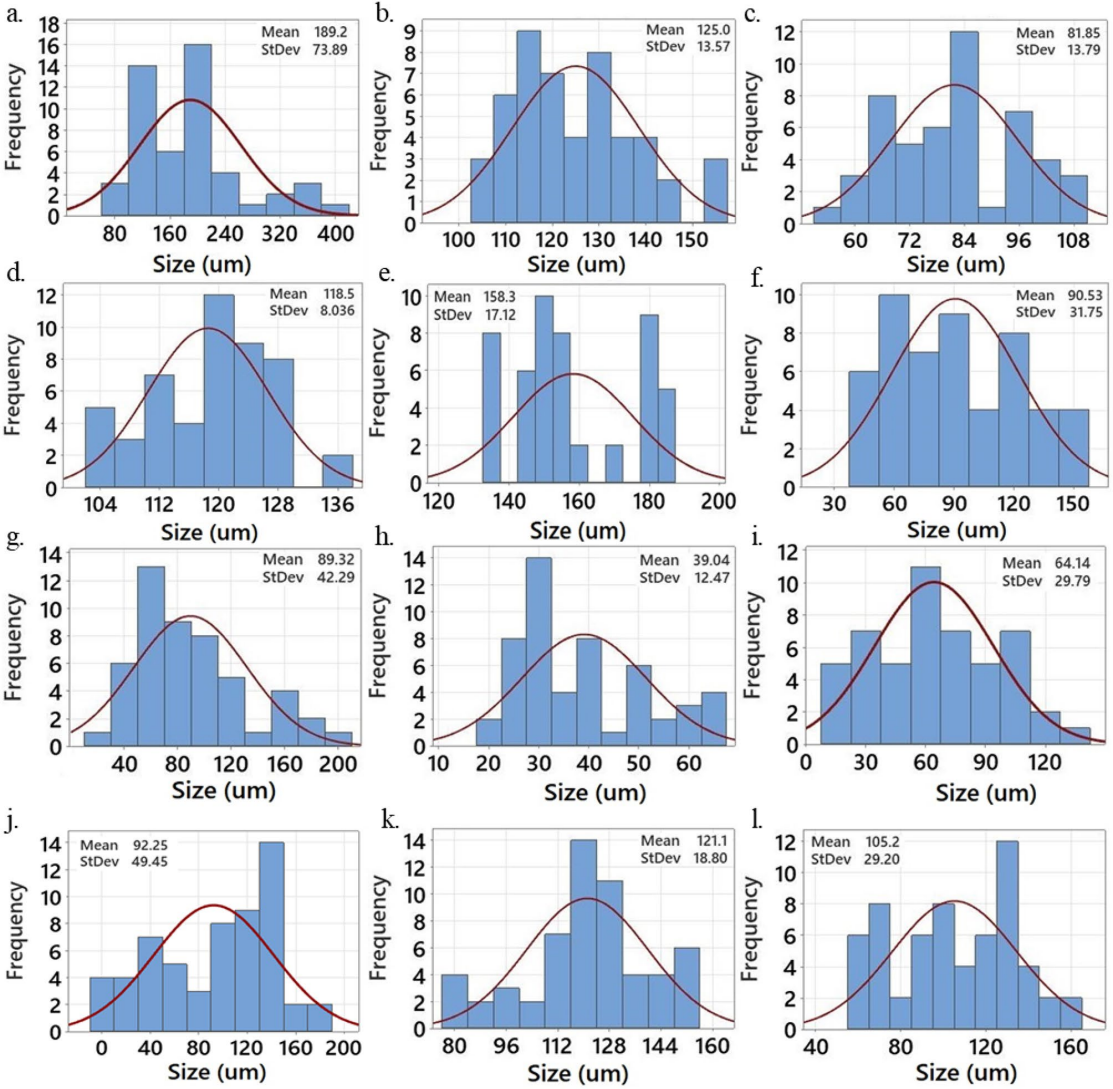
Cell size distribution of PLA foams: (**a**) Run 5, (**b**) Run 9, (**c**) Run 10, (**d**) Run 11, (**e**) Run 12, (**f**) Run 15, (**g**) Run 18, (**h**) Run 24, (**i**) Run 26, (**j**) Run 28, (**k**) Run 29, (**l**) Run 30.

The surface area is frequently determined using the BET method, which involves the application of the BET theory (Eq. 4) to the N_2_ adsorption isotherm.

4 $$\frac{1}{{\vartheta \left[ {\left( {P_{0} /P} \right) - 1} \right]}} = \frac{C - 1}{{\vartheta_{m} C}}\left( {\frac{P}{{P_{0} }}} \right) + \frac{1}{{\vartheta_{m} C}}$$

The highest specific surface area of foam is found to be 18.761 m^2^/g, which is in close agreement with previous investigation by Zhang et al.^41^.

### Effect of polymer concentration

**Figure 7:**
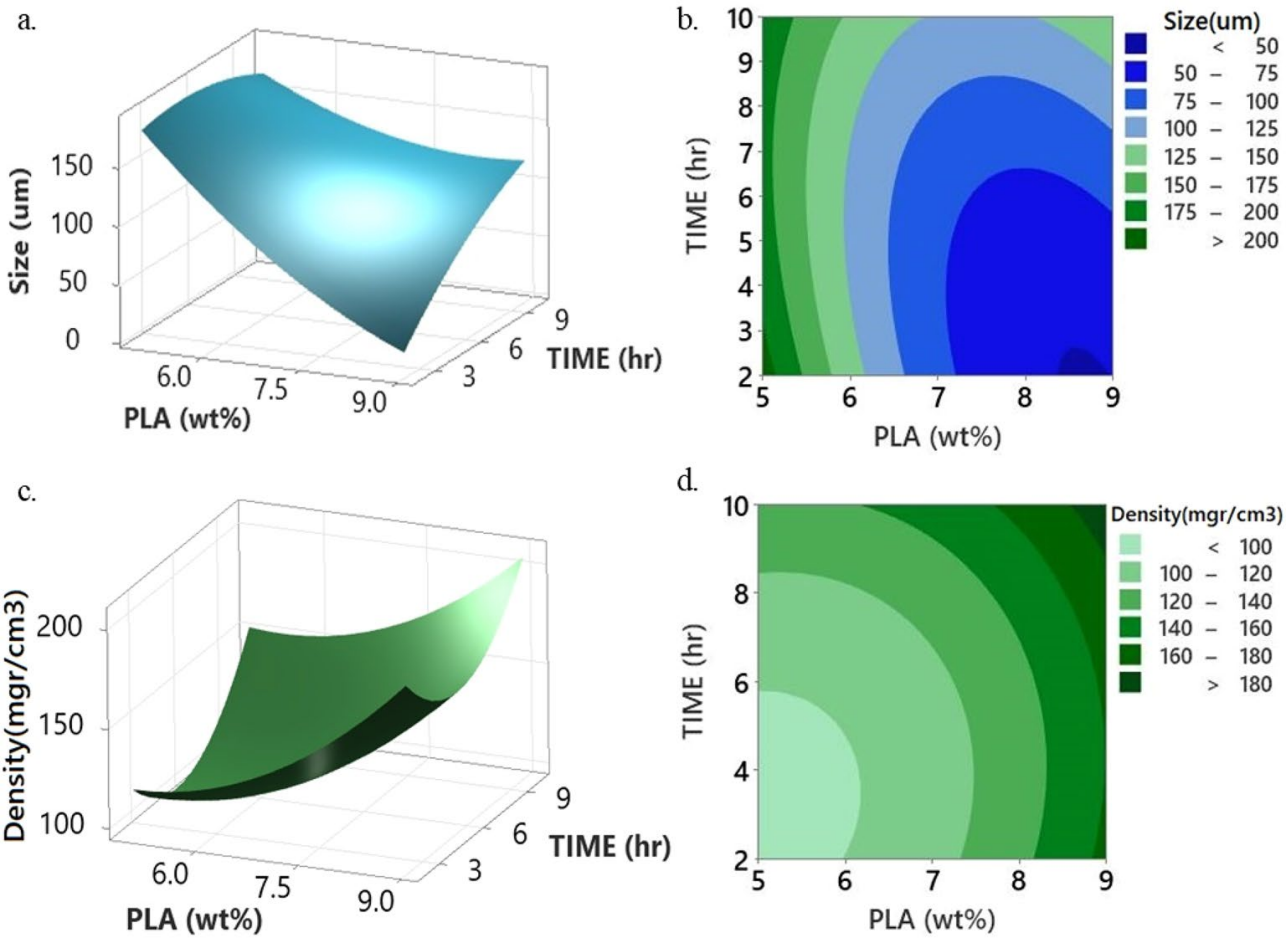
Plots of (**a**, **c**) response surface and (**b**, **d**) contour for the effect of PLA concentration and aging time on cell size and density at constant THF/water ratio and quenching temperature in central point.

Increasing the polymer concentration from 5 to 9 wt% caused a decrease in the interconnectivity between cellular pores and a reduced pore size throughout the foam. In the polymer-rich phase matrix, two or more droplets merge during contact to form a single droplet during coalescence process. As this network becomes coarser over time, solvent droplets include in the polymer-lean phase. The interfacial energy between polymer-rich and polymer-lean phases is minimized by the coalescence coarsening process. Upon gelation of the polymer-rich phase, this procedure is stopped. As the polymer content decreases, the kinetics of phase separation slow down, leading to an increase in gelation time. This prolonged duration allows for the coarsening process to occur, ultimately resulting in the creation of foams characterized by more giant (Fig. 7a).

An increase in the quantity of solvent results in a decrease in viscosity, consequently prolonging the time required for the substance to transition into a gel. As the duration of gelation is extended, there is a possibility of a prolonged occurrence of coarsening through the coalescence of solvent droplets in the polymer-lean phase. A decrease in viscosity results in the facilitation of the movement of solvent droplets, thus promoting rapid coalescence. The escalation of solvent quantity leads to a rise in the number density of droplets generated during the process of spinodal decomposition, consequently resulting in a reduction of the distance between droplets. It is hypothesized that the effects as mentioned above will lead to the conglomeration of droplets, which will increase the droplet size and subsequently enlarge the pore dimension after solvent movement.

The size of pores becomes more prominent when the amount of PLA in the solution decreases. The coarsening process may persist for extended durations with an increase in gelation time due to the merging of solvent droplets in the polymer-lean phase. The presence of a high concentration of polymers significantly impacts the viscosity of the polymer solution, thereby causing a decrease in the rate of the droplet growth rate in the foam. This reduced growth rate of droplets leads to a slower widening process of pore size, ultimately resulting in the formation of smaller pores. This finding agrees with the findings of Onder et al. in their investigation on the development of rigid polylactic acid foams^11^ as well as with the research conducted by Gay et al. on the production of PLA scaffolds^38^.

Increasing the polymer concentration causes to the creation of a denser foam structure (Fig. 7c). This phenomenon can be clarified by increasing the polymer concentration escalates the nucleation. The density of foam nucleation plays an essential role in reducing the interconnectivity between pores. So, this process leads to the blocking of the pores, ultimately resulting in a higher density, as shown in Fig. 7c. This finding is consistent with Othman's reports, which emphasize the significant impact of polymer concentration on the foam's morphology, especially with regard to the porosity of PLA foam fabricated using the TIPS technique^42^.

### Effect of aging time

Achieving control over morphology development during phase separation, under the specified thermodynamic driving force, is possible through the adjustment of aging time. Figure 7 illustrates the impact of aging time on cell size and foam density. This study was carried out at their central points when solvent composition and temperature were constant. The elevated aging time cause appropriate enough time for the phase separation and nuclei growth on interface led to the cell size enhancement.

The distinctive interconnected porous structure is identified during the initial stage of the process. This unique morphology plays a crucial role in determining the properties and performance of the material being studied. It is supposed that porous morphology results from the initial step of phase separation controlled by a spinodal decomposition mechanism. Overextended aging periods, polymer sedimentation occurs alongside the coarsening process. The gravitational force-induced sedimentation facilitates the creation of two separate regions: a solvent phase region and a polymer-rich phase region. The coarsening process leads to the formation of small porous structures within the polymer-rich phase, which can be attributed to the occurrence of secondary phase separation. The significance of this particular phenomenon plays a crucial role in determining the porous structure of the material being studied. The sedimentation of polymers triggers the generation of a foam that is poorly interconnected and characterized by irregular and closed pores^14,43,44^.

In addition, excessive aging time and high levels of cross-linking level caused PLA to agglomerate and block the foam pores. The occurrence resulted in a decrease in both the mean pore diameter and total porosity, consequently causing an increase in foam density, as observed in the PVDF membrane system by Sun et al.^45^.

### Effect of solvent composition

To determine the free energy of mixing in a polymer solution, the Flory–Huggins theory^44,46^ is utilized.

5 $$\frac{{{\text{f}}\left( {\emptyset \cdot T} \right)}}{{{\text{k}}_{B} {\text{T}}}} = \frac{\emptyset }{{\text{N}}}\ln \emptyset + \left( {1 - \emptyset } \right)\ln \left( {1 - \emptyset } \right) + \upchi \emptyset (1 - \emptyset )$$

where k_B_ is the Boltzmann constant, T is the absolute temperature, N is the average degree of polymerization, χ represents the Flory parameter, and φ is the polymer concentration.

The initial couple of components in the equation pertaining to the thermodynamic property of mixing exhibit an entropic characteristic, whereas the third term indicates the adequate contact energy. It is noteworthy that the entropic contribution is invariably negative, thereby facilitating the process of mixing. Nevertheless, its magnitude is relatively trivial and thus, can be considered as negligible. The final term in the sequence pertains to the enthalpic contribution, which is exclusively reliant upon the interaction parameter within a particular concentration of polymer. When this parameter (χ) is negative, a uniform solution will be achieved at a designated temperature. While the value of (χ) is positive, there is a tendency towards demixing which results in the occurrence of LLPS^47^.

In the case of a UCSD, the Flory parameter varies inversely with T.

6 $$\upchi = {\text{z}}\Delta \upvarepsilon /{\text{k}}_{\text{b}} {\text{T}}$$

7 $$\Delta \upvarepsilon = \upvarepsilon_{ps} - (\upvarepsilon_{pp} + \upvarepsilon_{ss} )/2$$

In the context of the formula, Z stands for the coordination number, Δε indicates the disparity in bond energy, ${\upvarepsilon }_{\text{ps}}$ is polymer–solvent interaction energy, ${\upvarepsilon }_{\text{pp}}$ is polymer–polymer interaction energy and ${\upvarepsilon }_{\text{ss}}$ solvent–solvent interaction energy. When Δε is < 0, PS contacts are favoured and for Δε > 0, PP & SS contacts are preferred.

To induce LLPS at operationally feasible temperatures, water must be added as a non-solvent for PLA in the THF solvent system. Hence, the THF/water ratio, an imperative factor in ascertaining the solvent quality, assumes pivotal functions in the kinetics as well as thermodynamics of the LLPS.

**Figure 8:**
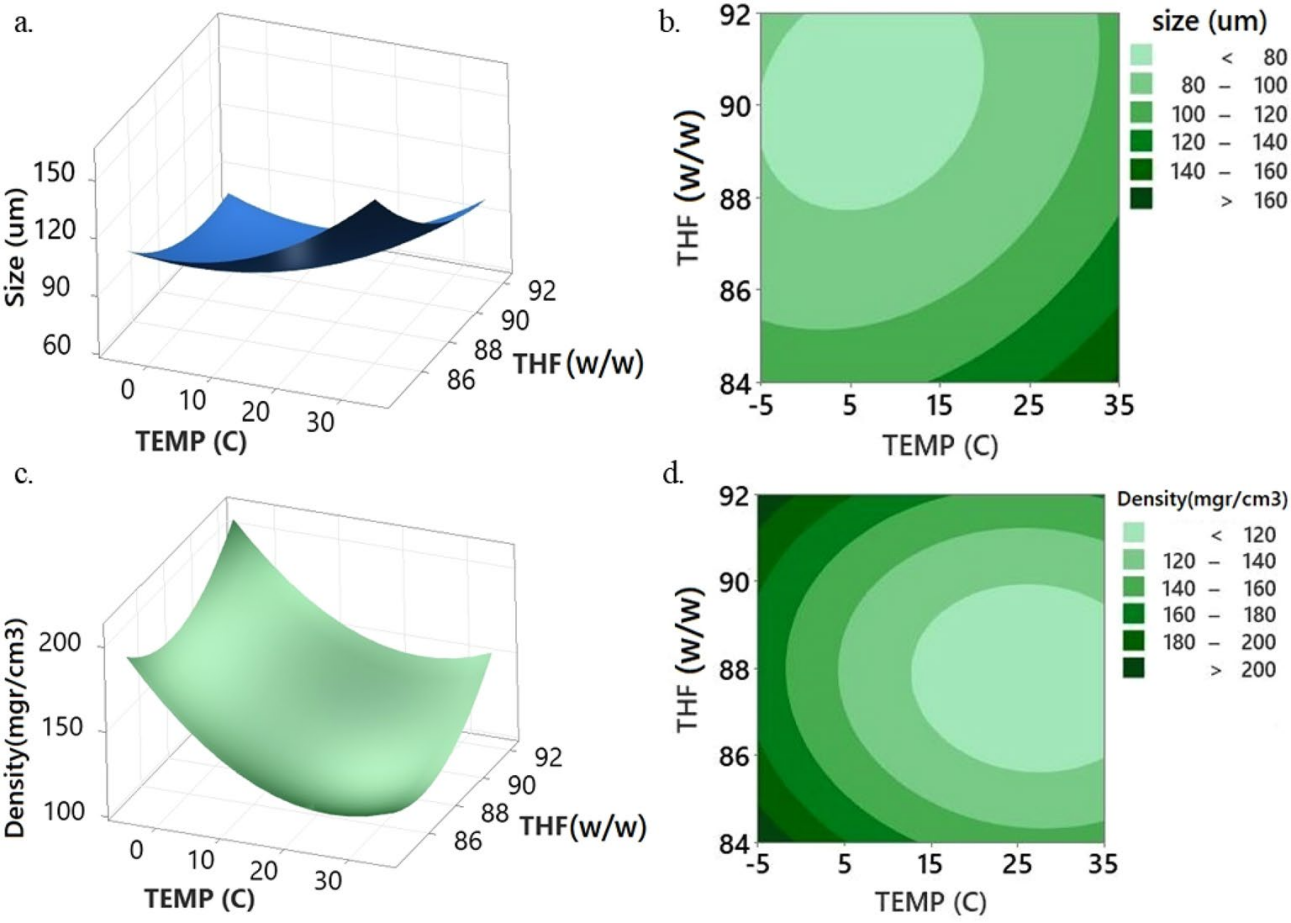
Plots of (**a**, **c**) response surface and (**b**, **d**) contour for the effect of quenching temperature and THF/water ratio on cell size and density at constant PLA concentration and aging time in central point.

**Figure 9:**
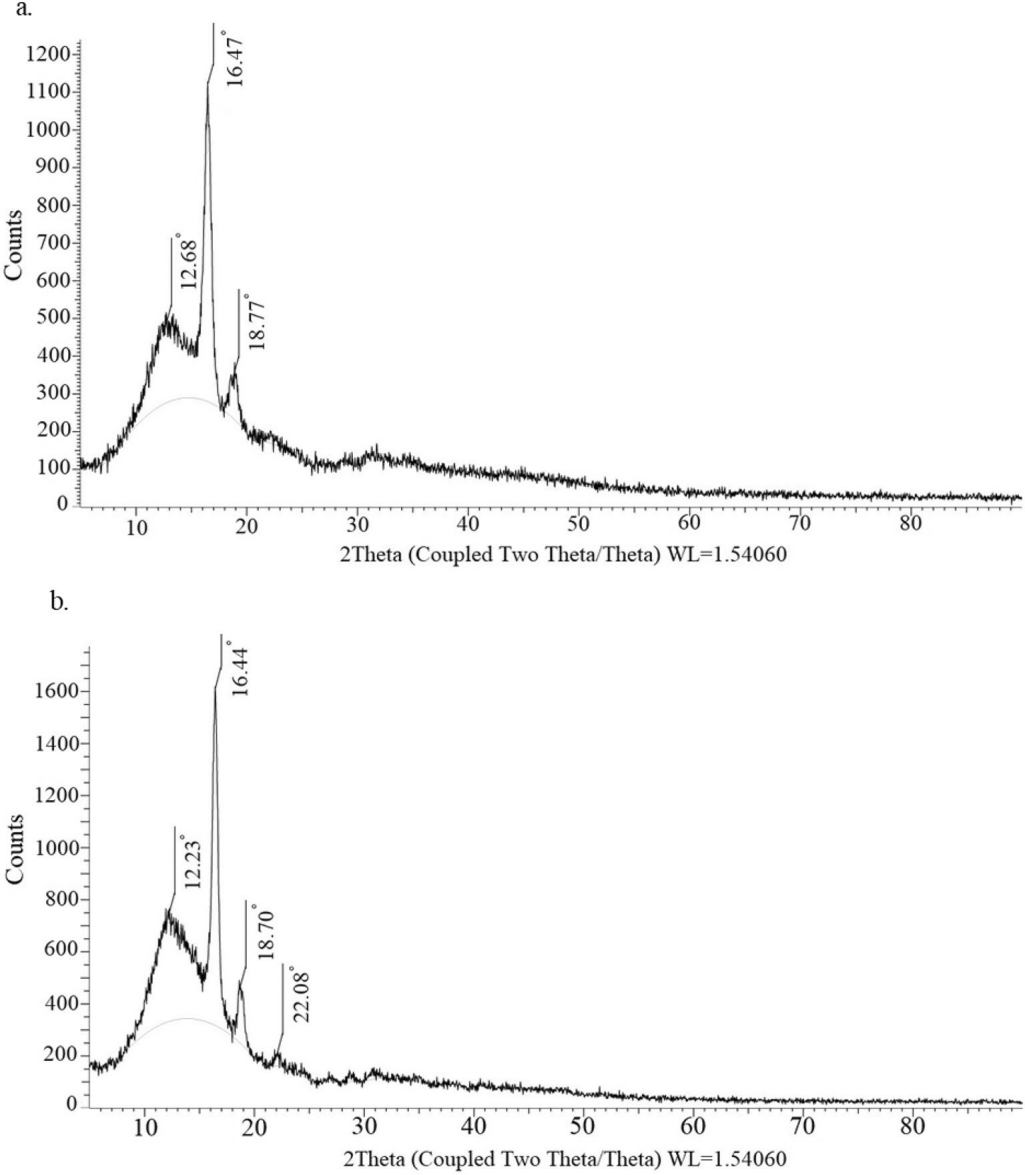
XRD diffractograms of polylactic acid foam (**a**) THF/water ratio 86/14 (w/w) and (**b**) THF/water ratio 90/10 (w/w).

A significant alteration from a macroporous morphology to a semi-nodular or globular occurs when the non-solvent content in the mixture reduces, resulting in a considerable decrease in pore sizes (Fig. 8a). The efficiency of the solvent is greatly improved as the percentage of water in the solute decreases from 16 to 8%. Consequently, there is a notable decrease in the cloud point. A spherical structure is observed distinctly when the ratio of THF/water is 92/8, as illustrated in Fig. 5j. Foam densities demonstrate a progressive decrease with the augmentation of water content. Correspondingly, the percentage porosity of the resulting foams experiences a slight increment. As previously stated, it is worth noting that the average pore size demonstrates a gradual reduction concomitant with a decline in the water content. The XRD measurements demonstrate a marginal rise in the crystallinity percentage values of the foams as the non-solvent content in the mixture decreases (Fig. 9). The increase in the THF/water ratio from 86 to 90 (w/w) leads to a corresponding increase in the percentage of crystallinity from 11.06% to 16.42%. The distinctive peaks of PLA foam were noted at 2θ = 12.68°, 16.47°, 18.77° (THF/water ratio 86/14 w/w), while four peaks related to the orthorhombic semi-crystalline structure were identified at 2θ = 12.23°, 16.44°, 18.70°, and 22.08° (THF/water ratio 90/10 w/w). Elevating the solvent's efficiency has the potential to expedite the mobility of the polymer chain in rich phase. This subsequently culminates in the manifestation of a greater degree of crystallinity. By raising the degree of crystallinity, the material's density is increased^48^. The density of the foams can be ascertained by evaluating the density values of the crystalline and amorphous phases of PLA, as per the provided equations^5^. In this equation, X_C_ represents the percentage of crystallinity, D_a_ signifies the density of the amorphous phase, and D_C_ denotes the density of the crystalline phase

8 $${D}_{PLA}=\frac{1}{\frac{1-{X}_{C}}{{D}_{a}}+\frac{{X}_{C}}{{D}_{C}}}$$

### Effect of quench temperature

The temperature adjustment during the quenching plays a vital role in both the kinetics and thermodynamics of phase separation and crystallization phenomena, thus serving as a key determinant in shaping the morphologies of the final foams. During the initial stage of the quenching process, the system undergoes phase separation characterized by a metastable region that is governed by the mechanism of nucleation and growth. Consequently, the system proceeds towards the unstable region where the spinodal decomposition occurs. Rapid cooling of the system may result in a brief period of metastability before quickly transitioning into the unstable region. Accordingly, one can assert that spinodal decomposition serves as the dominant mechanism for phase separation in a THF/water mixture.

Figure 8 displays the impacts of temperature on the cell size and density at the fixed PLA concentration and aging time of 7% and 6 h, respectively. The polymer-rich phase experiences a delay in gelation as the quenching temperature increases. The coarsening process, which is assumed to be prolonged, facilitates the merging of solvent droplets in the polymer-lean phase. As a consequence of solvent removal, there is an observed increase in droplet size and the formation of more giant cells. As the temperature of the quenching process is elevated from − 5 to 35 °C, a discernible decrease in the density of the obtained foams can be observed. This reduction in density is accompanied by a corresponding increase in the percentage of porosity. The observed phenomenon is likely attributed to a decelerated phase separation and crystallization process of PLA upon elevation of the temperature in the solution. As illustrated in Fig. 5g and d, by quenching a 7% polylactic acid solution in a THF/water mixture (88/12) and aging time of 6 h at different temperatures − 5 °C and 35 °C, a homogeneous foam morphology and highly viscous gel with spherical morphology were achieved, respectively. The investigation shows that the crystallization of PLA was impeded at high quenching temperatures due to the nucleation and growth process, resulting in the absence of foam formation. The effects of quenching temperature on foam morphology have been discussed by Akbarzadeh et al., Zhuo et al., and Nam et al.^49–51^.

### Model fitting and experimental data analysis

After excluding insignificant terms, the Minitab 21 software generated fitted models for density and cell size, which are presented in Eqs. (9) and (10). The coefficients shown in each equation serve to illustrate the influence of the corresponding parameter on the specified property. It is essential to note that the positive coefficients in the linear model contribute to a synergistic effect on the responses, whereas the negative coefficients result in a diminishing impact^52^.

9 $$\begin{aligned}Size & = 82.94-25.98*{y}_{1}-10.90*{y}_{2}+8.63*{y}_{3}+10.14*{y}_{4}+12.19*{{y}_{1}}^{2}+4.22*{{y}_{2}}^{2}+4.29*{{y}_{3}}^{2}\\ &\quad+4.97*{{y}_{3}}^{2}-5.82*{y}_{1}*{y}_{2}+7.25*{y}_{1}*{y}_{4}-5.03*{y}_{2}*{y}_{4}+4.97*{y}_{3}*{y}_{4}\end{aligned}$$

10 $$\begin{aligned}Density & = 116.16+15.60*{y}_{1}-15.20*{y}_{3}+8.70*{y}_{4}+4.05*{{y}_{1}}^{2}+11.07*{{y}_{2}}^{2}+6.46*{{y}_{3}}^{2}\\ &\quad+3.81*{{y}_{4}}^{2}+16.16*{y}_{1}*{y}_{2}-3.74*{y}_{1}*{y}_{3}-4.88*{y}_{3}*{y}_{4}\end{aligned}$$

The validation process of the experimentally derived RSM model included the application of several statistical parameters. The current research paper evaluates the impact of each factor in the mathematical model by utilizing the *p*-value and f-value. A lower *p*-value indicates a more significant model. Typically, a *p*-value of 0.05 or less is considered to be adequate. The effectiveness of the quadric model's suitability was evaluated by employing both the multiple correlation coefficient (R^2^) and the "lack of fit (LOF)" parameter. If the *P*-value for the lack of fit is above 0.05, it indicates that the LOF is not statistically significant. In other words, when the *p*-value is exceeds the chosen significance level, the test fails to detect any evidence of lack-of-fit. These results indicate that the proposed model accurately represented the experimental measurements^53^. The regression equations generated by analysis of variance (ANOVA) demonstrate strong accuracy with R^2^ and R^2^ (adj.) values 95.68%, and 91.90%, respectively for cell size and also 96.09%, and 92.68% for density. The *p*-values for LOF regarding cell size and density were found to be 0.328 and 0.212, respectively. The identified values have been determined to be significantly above the threshold of 0.05^30^. The coefficients of multiple regression and the outcome of the second-order response surface model, presented through the ANOVA format for both cell size and density, are displayed in Tables 4, 5.

**Table 4:** Regression coefficients and (ANOVA) of the response surface quadric model for cell size.

Term/source	Coef	SE Coef	Adj. SS	Adj. MS	T-value	*P*-value	F-value	Cont. (%)
Model			30801.6	2200.1		0.000	25.32	
Linear			23304.6	5826.2		0.000	67.06	
Constant	82.94	3.52			23.54	0.000		
y_1_	− 25.98	1.90	16195.9	16195.9	− 13.65	0.000	186.42	50.31
y_2_	− 10.90	1.90	2851.9	2851.9	− 5.73	0.000	32.83	8.86
y_3_	8.63	1.90	1787.1	1787.1	4.54	0.000	20.57	5.55
y_4_	10.14	1.90	2469.7	2469.7	5.33	0.000	28.43	7.67
Square			5003.4	1250.9		0.000	14.40	
y_1_^2^	12.19	1.74	4247.9	4247.9	6.99	0.000	48.89	13.20
y_2_^2^	4.22	1.74	509.4	509.4	2.42	0.028	5.86	1.58
y_3_^2^	4.29	1.74	526.7	526.7	2.46	0.026	6.06	1.64
y_4_^2^	4.97	1.74	707.2	707.2	2.85	0.012	8.14	2.20
2-way Interaction			2493.6	415.6		0.006	4.78	
y_1_*y_2_	− 5.82	2.33	541.5	541.5	− 2.50	0.024	6.23	1.68
y_1_*y_3_	4.03	2.33	260.3	260.3	1.73	0.103	3.00	0.81
y_1_*y_4_	7.25	2.33	841.0	841.0	3.11	0.007	9.68	2.61
y_2_*y_3_	− 1.78	2.33	50.9	50.9	− 0.77	0.455	0.59	0.16
y_2_*y_4_	− 5.03	2.33	404.4	404.4	− 2.16	0.047	4.65	1.26
y_3_*y_4_	4.97	2.33	395.4	395.4	2.13	0.049	4.55	1.23
Error			1390.1	86.9				
Lack-of-fit			988.6	98.9		0.328	1.48	
Pure Error			401.5	66.9				1.25
Total			32191.7					100

**Table 5:** Regression coefficients and (ANOVA) of the response surface quadric model for density.

Term/Source	Coef	SE Coef	Adj. SS	Adj. MS	T-value	*P*-value	F-value	Count. (%)
Model			19171.9	1369.42		0.000	28.11	
Linear			13257.4	3314.36		0.000	68.04	
Constant	116.16	2.64			44.03	0.000		
y_1_	15.60	1.42	5838.1	5838.14	10.95	0.000	119.85	28.68
y_2_	1.57	1.42	59.4	59.41	1.10	0.286	1.22	0.29
y_3_	− 15.20	1.42	5544.4	5544.35	− 10.67	0.000	113.82	27.24
y_4_	8.70	1.42	1815.5	1815.52	6.10	0.000	37.27	8.92
Square			4584.4	1146.09		0.000	23.53	
y_1_^2^	4.05	1.31	469.5	469.5	3.10	0.007	9.64	2.31
y_2_^2^	11.07	1.31	3505.5	3505.47	8.48	0.000	71.96	17.22
y_3_^2^	6.46	1.31	1194.5	1194.51	4.95	0.000	24.52	5.87
y_4_^2^	3.81	1.31	414.2	414.15	2.92	0.010	8.50	2.03
2-way Interaction			1330.1	221.69		0.006	4.55	
y_1_*y_2_	6.19	1.74	613.6	613.55	3.55	0.003	12.60	3.01
y_1_*y_3_	− 3.74	1.74	223.8	223.8	− 2.14	0.048	4.59	1.10
y_1_*y_4_	− 1.06	1.74	17.9	17.9	− 0.61	0.553	0.37	0.09
y_2_*y_3_	0.88	1.74	12.3	12.3	0.50	0.622	0.25	0.06
y_2_*y_4_	2.26	1.74	81.9	81.9	1.30	0.213	1.68	0.40
y_3_*y_4_	− 4.88	1.74	380.6	380.6	− 2.80	0.013	7.81	1.87
Error			779.4	48.71				
Lack-of-fit			596.5	59.65		0.212	1.96	
Pure Error			182.9	30.48				0.90
Total			19951.3					100

In ANOVA methodology the quantification of the impact of each factor on the response is then determined by evaluating the percentage contribution of each factor. As demonstrated in Tables 4, 5, PLA concentration with contribution percentage of 50.31% and 28.68% is the most effective parameter influencing cell size and density, respectively^54,55^.

The contribution of factor y was calculated using as follows:

11 $$contribution (\text{\%})=\frac{{SS}_{y}}{{SS}_{T}}$$

Where total sum of square denote as SS_T_, and SS_y_ represents sum of square of factor y.

**Figure 10:**
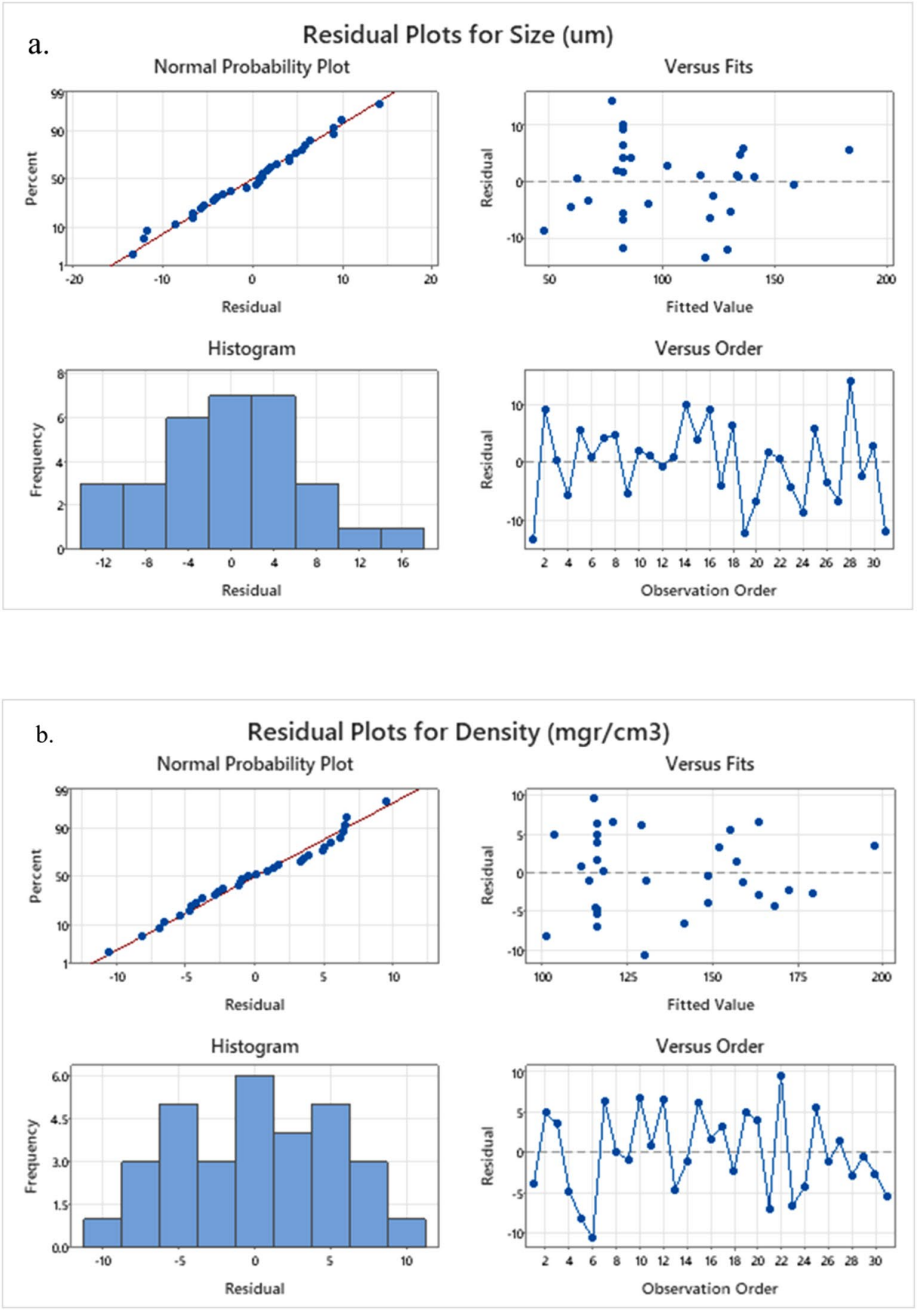
Different residual plots for testing the adequacy of the proposed model (**a**) cell size and (**b**) density.

As illustrated in Fig. 10 by utilizing graphical displays of the residuals, the model diagnostics process is completed. In the normal probability plot, the residuals indicate that the data points are closely clustered together, and follow a near-linear trend. Moreover, the histogram displays an almost symmetrical shape, resembling a normal distribution with minor deviations. Additionally, the residuals exhibit a consistent variance and a non-linear relationship as they are randomly dispersed around zero in the residuals versus fitted values plot. Moreover, the residual versus order plot does not display any discernible pattern, indicating the absence of any undesirable effects. Furthermore, Four in one residual plots do not reveal any anomalies for the fitted regression model.

**Figure 11:**
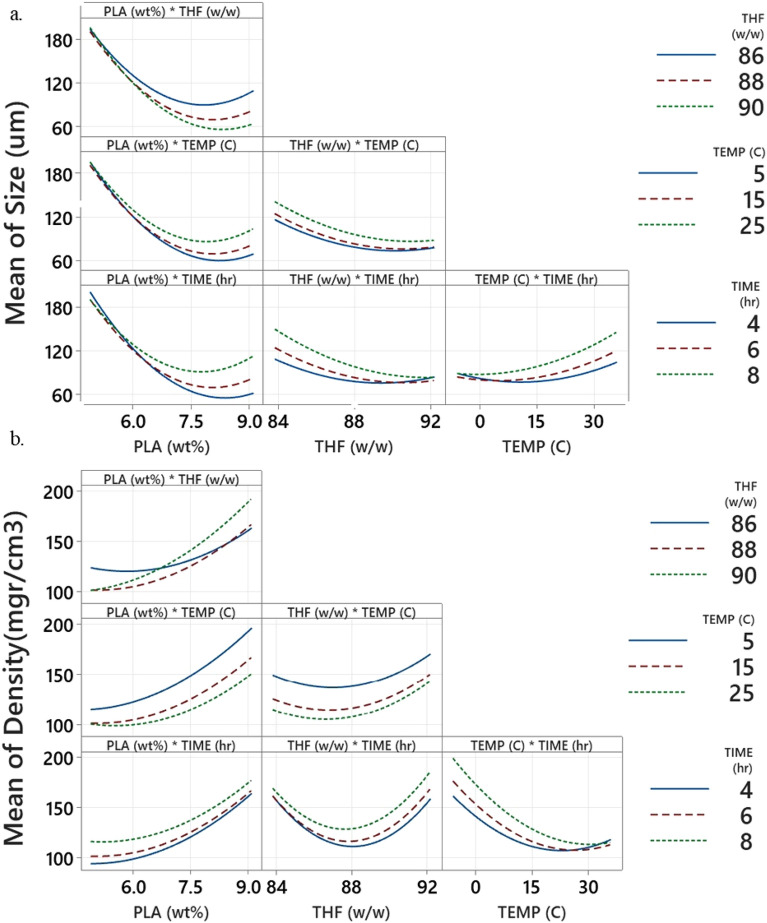
Interaction plot of the (**a**) cell size and (**b**) density.

The individual factors and their interactions have different effects on a response (cell size and density) are shown in Fig. 11. The DOE interaction effects plot allows us to draw several conclusions. Figure 11 illustrates the varying impacts of individual factors and their interactions on a response (specifically cell size and density). By examining the DOE interaction effects plot, researchers are able to discern several key conclusions regarding how these factors influence the outcome. Firstly, they can identify the crucial factors by examining the plots with the steepest lines, indicating the most considerable effects. Secondly, they can determine the best settings to enhance sensitivity. The non-parallel lines contribute to a notable interaction between these elements^56,57^. The results indicate that y_1_*y_3_ and y_2_*y_3_ are not significant factors influencing cell size, whereas y_1_*y_4_, y_2_*y_3_, and y_2_*y_4_ do not play a significant role in determining density.

It was noticed that the polymer concentration increased (in constant other variables), and the pore size of the foam decreased while density increased. Analysis of the interaction effect curves indicated that at elevated levels of THF/water ratio, the impact of non-solvent reduction on foam density was amplified. Moreover, with a decrease in the amount of non-solvent, the foam's cell size was reduced.

The graph provides a visual depiction of the relationship between temperature and time. It is apparent that there is a broader distribution of points on the right side of the graph, corresponding to higher temperatures, as opposed to the left side, where temperatures are lower. This disparity indicates that the influence of time is more prominent at elevated temperatures. Consequently, exposure to extremely low temperatures may mitigate the impact of reaction time, resulting in a reduction in cell size. Furthermore, an overall trend of increasing cell size with prolonged exposure time is evident. Additionally, when the quenching temperature is increased, resulting in an increase in cell size and decrease density.

### RSM and genetic algorithm optimization

Genetic algorithms (GA) have emerged as a highly effective optimization method with several notable advantages: (I) they do not require the objective function to possess continuity, convexity or unimodality, and (II) they are able to execute parallel searches in the viable space and test small blocks of effective solutions across multiple situations, these entities demonstrate a remarkable level of efficiency^58^. Advantages as mentioned earlier, make them exceptionally well-suited for optimization in RSM, mainly in scenarios involving discontinuity or when spaces are highly constrained or irregularly configured. GA has proven to be highly productive in the optimization of response variables, as well as in multi-response scenarios. The optimization of the Response Surface can be achieved through the utilization of a genetic algorithm with the objective to identifying the optimal values for the independent variables. The outline below encapsulates the process by which the genetic algorithm operates^59,60^:

1. Start: The algorithm begins by generating an initial population, which is randomly chosen and sized based on empirical observations. For this particular study, a population size of 100 was chosen.
2. Fitness: This stage involves the assessment of the fitness f(x) of each chromosome in concerning the specific problem, determining their level of suitability.
3. New population: The process of generating a new population, also known as a generation, involves the selecting the fit chromosomes for mating.
  - Selection: The selection process for chromosomes prioritizes those with superior fitness, increasing the probability of their choice as mates. (Selection method: tournament).
  - Crossover: Through the crossover function, a new chromosome is formed by combining portions of two parent chromosomes via splicing. (Crossover method: two point crossover, crossover fraction: 0.6).
  - Mutation: Similar to the random mutations found in nature, the mutation function is responsible for preserving genetic diversity among populations. Through modifying gene values, it contributes to the improvement of offspring quality. (Mutation method: Gaussian).
  - Replace: Upon generation, the novel population will substitute the existing population.
4. It is necessary to repeat steps 2 and 3 until the termination conditions are satisfied. These conditions can be fulfilled when either the specified value of iterations is achieved or the fitness function satisfies the tolerance criteria. In this particular scenario, the selected values for these conditions are 100 and 10^−4^, respectively.

**Figure 12:**
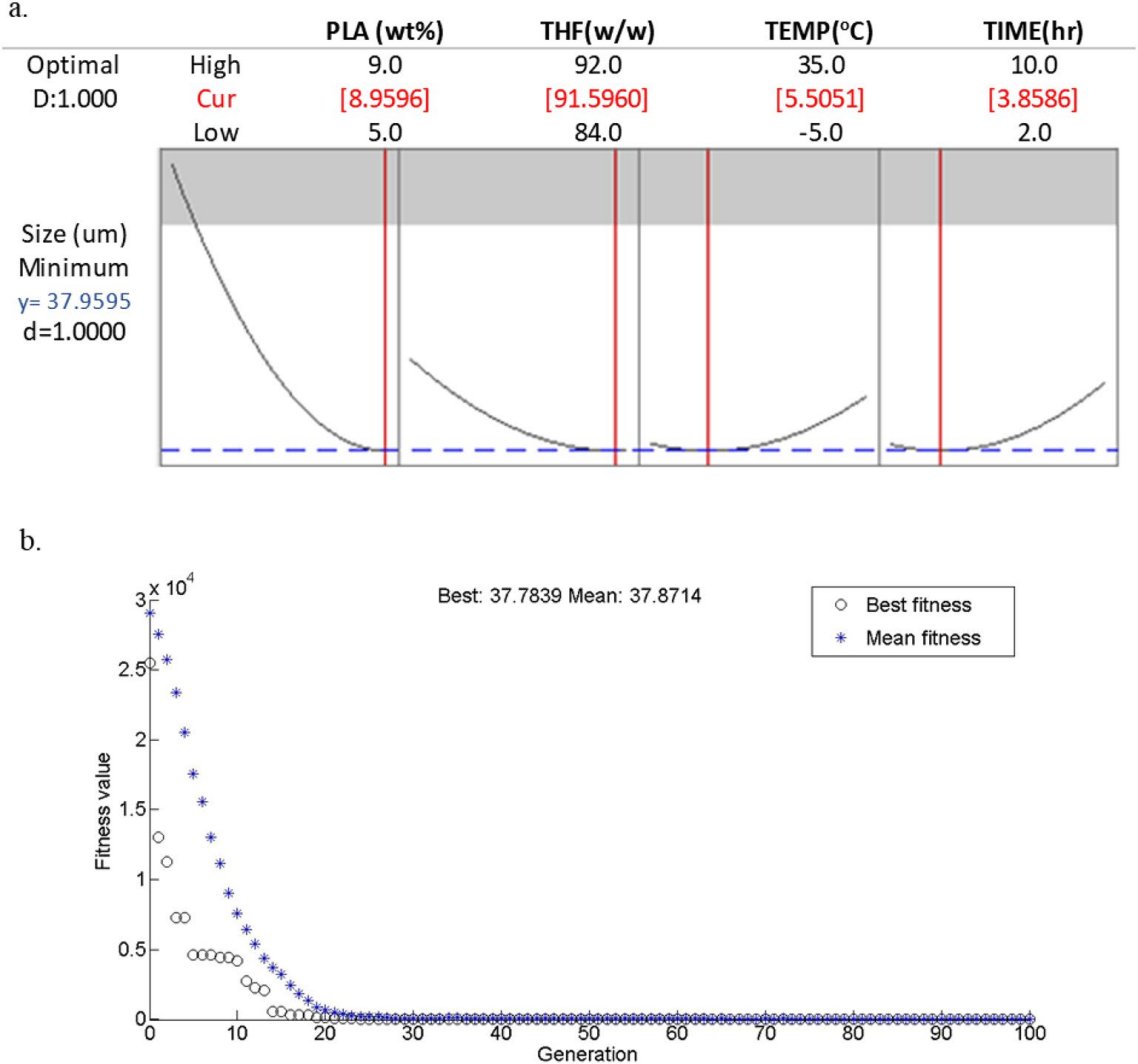
(**a**) Optimization plot for cell size and (**b**) Variation of fitness function versus generation.

**Figure 13:**
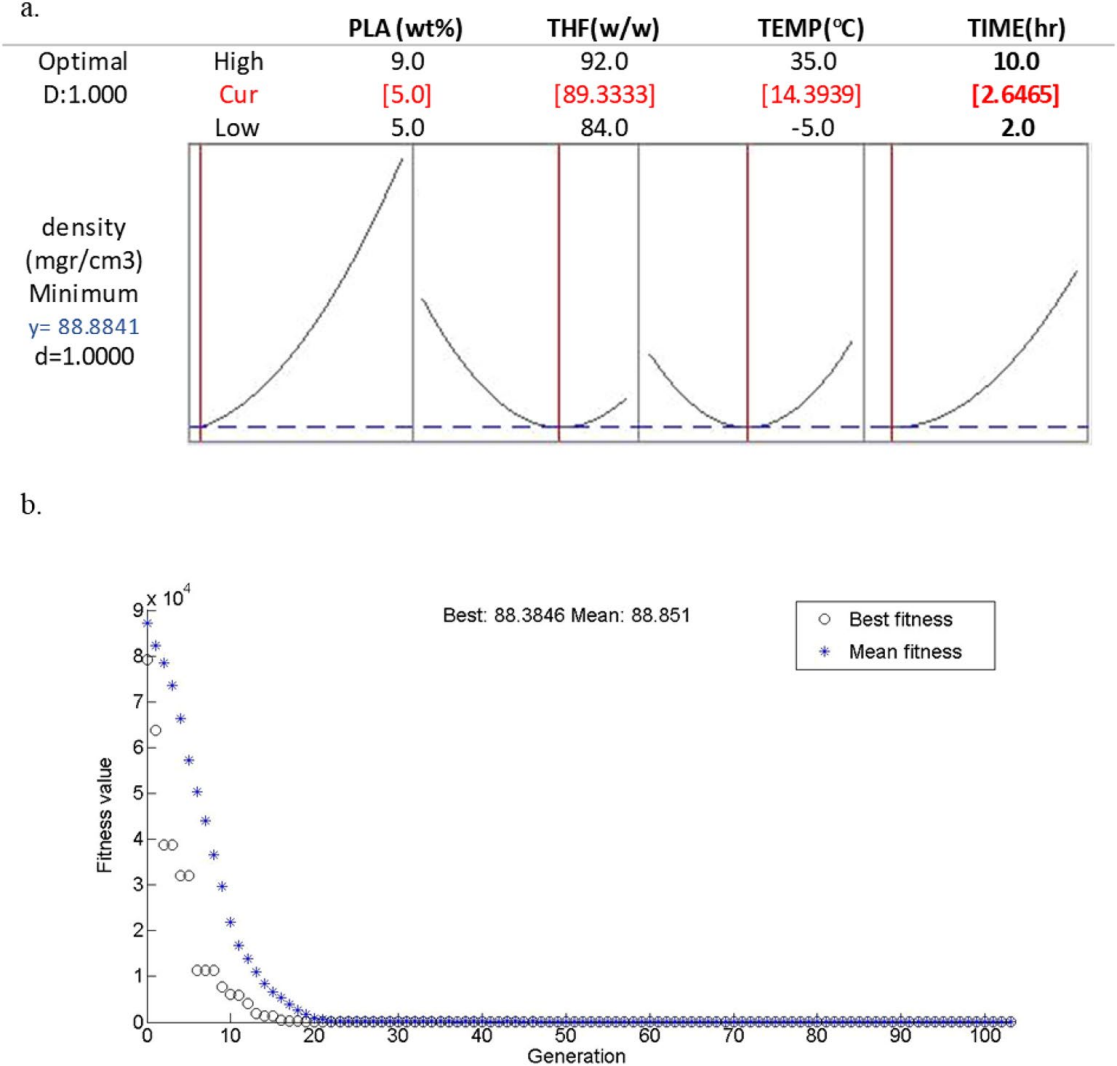
(**a**) Optimization plot for density and (**b**) Variation of fitness function versus generation.

The RSM methodology was employed to conduct optimization, aiming to achieve the best responses, which include minimum cell size and density. The obtained results are presented in Figs. 12, 13. Under the accepted optimal condition, the values of the cell size are 8.96 wt%, 91.60 w/w, 5.50 °C, and 3.86 h with the predicted cell size 37.96 µm by RSM and 37.78 µm by GA. Furthermore, the outcomes displayed that the optimum density for foam were as monitors: PLA concentration of 5.00 wt%, solvent composition of 89.33 w/w, quench temperature of 14.40 °C and aging time of 2.65 h with the predicted density of 88.88 (mgr/cm^3^) by RSM and 88.38 (mgr/cm^3^) by GA. The precision and accuracy of the developed model were assessed through the implementation of statistical analysis, which demonstrated a strong concordance between the observed results and the mathematical model.

The genetic algorithm offers an optimal selection of extracted features that can be effectively utilized alongside the individual classifiers. The variation of fitness value (cell size and density) versus generation is presented in Figs. 12b and 13b. The given figure visually represents the gradual improvement of mean fitness across successive generations, ultimately converging towards the best fitness level. This suggests that the population comprises an adequate number of the best individual copies, obviating the need for further exploration.

### Water absorption

The water absorption test was performed following the Cobb method as outlined in NBR NM ISO 535:1999, which determines the Water Absorption Capacity (WAC) of polymers after being immersed. The presented methodology entails the determination of the escalation in the mass of the sample after to its direct contact with water^61,62^. Briefly, previously weighed samples floated in 10 mL of distilled water for 24 h. After the removal of excess water via the utilization of tissue paper, the PLA foams were once again subjected to weighing. The foam's water absorption capacity was evaluated by measuring the amount of water (in grams) absorbed per grams of the original sample^63^. The experiments were performed with a minimum of three replicates. WAC was calculated by :

12 $$WAC = \left( {M_{2} - M_{1} } \right)/M_{1}$$

**Figure 14:**
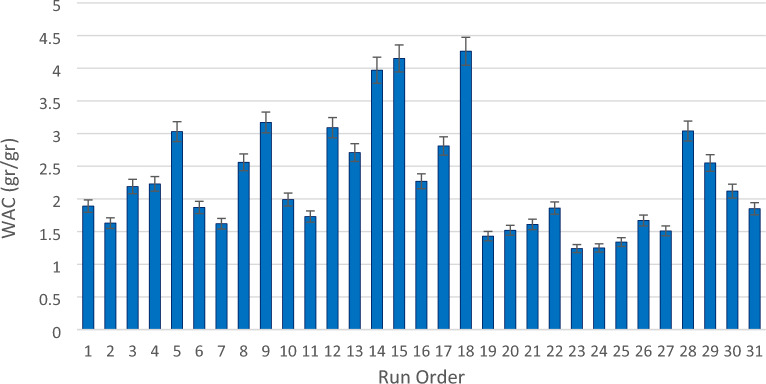
Water absorption capacity of the PLA foams.

The parameters M_1_ and M_2_ are utilized to represent the weight of the foam pre-sorption and post-sorption, respectively. Figure 14 shows the water absorption capacity of the PLA foams. The depicted result showcases the maximum capacity achieved, which was obtained through the sampling of number 18. The experimental data obtained in this investigation exhibits a high level of compatibility with the reported results from a similar examination conducted by Li et al.^64^. According to this report, the open-cell PLA foams have been found to possess remarkable oil-sorption properties. This finding not only presents a novel approach for the sustainable creation of bio-based and biodegradable sorption foam but also emphasizes its capacity to effectively absorbing oil, dye, and heavy metals from aqueous solutions.

## Conclusions

Rigid foams of PLA were procured utilizing thermally induced phase separation. In this study, a comprehensive analysis was carried out to understand how polymer concentration, solvent composition, quench temperature, and aging time affect the foaming procedure, as well as the characteristics of the samples obtained. The regulation of foam densities and average pore sizes was primarily achieved by adjusting of these variables. In general, PLA foams exhibiting a relatively high degree of porosity, ranging from 83.76% to 92.5%, were successfully produced. Foams having pore dimensions ranging from 39 to 189 µm were successfully generated. The results of this investigation showed that the quadratic polynomial model effectively characterized and forecasted the alterations in cell size and density caused by variations in the operating conditions of the TIPS procedure.

The Developed model predicted that the optimal conditions for cell size would be 8.96 wt %, 91.60 w/w, 5.50 °C, and 3.86 h. RSM forecasted a cell size of 37.96 µm, while the GA predicted a cell size of 37.78 µm. These results indicate that the model was able to accurately determine the ideal parameters for achieving the desired cell size. Furthermore, the outcomes exhibited that the optimum conditions for density were as monitors: PLA concentration of 5.00 (wt%), solvent composition 89.33 (w/w), quenching temperature of 14.40 °C and aging time of 2.65 h with the RSM predicted density of 88.88 (mgr/cm^3^) and GA predicted 88.38 (mgr/cm^3^), respectively.

## Data Availability

Data sets generated during the current study are available from the corresponding author on reasonable request.
